# Steroid pulse therapy for VKH during pregnancy: a safe and effective option?

**DOI:** 10.1186/s13023-025-03916-9

**Published:** 2025-07-16

**Authors:** Umashri Sundararaju, Shanmathi Subramanian, Hamrish Kumar Rajakumar

**Affiliations:** Government Medical College, Omandurar Government Estate, Chennai– 02, Tamilnadu India

## Abstract

Vogt–Koyanagi–Harada syndrome (VKH) is a rare autoimmune condition affecting melanocyte-containing tissues such as the eyes, skin, hair, inner ear, and meninges. The disease progresses through four phases: prodromal, uveitic, convalescent, and recurrent. Symptoms include headaches, photophobia, ocular inflammation, and auditory disturbances. Although the etiology behind VKH is unknown, it is believed to be triggered by viral infections such as the Epstein‒Barr virus or Cytomegalovirus in genetically predisposed individuals. It is diagnosed by confirming bilateral ocular involvement and ruling out other conditions, such as tuberculosis. It is managed by corticosteroids, with steroid pulse therapy being effective for controlling inflammation and improving outcomes. However, it carries significant risks, such as hypertension, infections, and electrolyte imbalances. Managing VKH during pregnancy can be challenging and requires a multidisciplinary approach, as corticosteroid use can affect both maternal and fetal health. Steroid therapy is crucial for controlling disease, but it can lead to complications such as infections, fetal growth restriction, and potential neurodevelopmental issues in infants. Future research should focus on optimizing steroid regimens, understanding the long-term effects of corticosteroid use during pregnancy, and developing personalized treatment strategies to improve maternal and fetal outcomes in VKH management.

## Introduction to VKH

Vogt–Koyanagi–Harada syndrome (VKH) is a rare systemic condition affecting melanocyte-containing tissues such as the eyes, inner ear, meninges, skin, and hair. It is characterized by intraocular inflammation along with associated abnormal cutaneous, auditory, and neurological manifestations [1]. The exact etiology of VKH remains uncertain, but it is commonly attributed to autoimmunity-targeting antigens related to melanocytes, which can be triggered by viral infections such as Epstein‒Barr virus or cytomegalovirus in genetically predisposed individuals [2, 3].

The clinical progression of VKH can be divided into four distinct phases: prodromal, uveitic, convalescent, and recurrent. In the prodromal phase, patients experience headache, vertigo, photophobia, and tinnitus. During the uveitic phase, patients typically present with bilateral posterior uveitis, decreased vision, and dysacusis. The convalescent phase is characterized by vitiligo, poliosis, and depigmentation of the choroid. The recurrent phase involves ongoing panuveitis with episodes of granulomatous anterior uveitis [4].

The diagnostic criteria for VKH disease include no preceding ocular trauma or surgery, bilateral involvement confirmed by indocyanine green angiography (ICGA) and/or enhanced depth imaging optical coherence tomography (EDI-OCT), the exclusion of other conditions such as tuberculosis or sarcoidosis, diffuse choroiditis, symptoms lasting less than 4 weeks, and the absence of chronic disease signs such as vitiligo. Additional criteria include exudative retinal detachment, disc hyperfluorescence, and abnormal neurological or auditory findings [5].

The primary treatment strategy for both pregnant and nonpregnant patients involves the early and aggressive use of systemic corticosteroids such as oral prednisone (100 to 200 mg) followed by gradual tapering over six months. It aims to suppress intraocular inflammation and its associated complications. In addition, nonsteroidal immunosuppressive agents such as cyclosporine (5 mg/kg/day) are important during the early stages of the disease. In cases where this regimen is insufficient, biologic therapies such as anti-TNF agents or the B-cell inhibitor rituximab can be used [4]. Table 1 summarizes the benefits and risks of various treatment approaches for VKH [4, 6].

**Table 1:** Overview of treatment approaches for Vogt–Koyanagi–Harada syndrome

Treatment	Benefits	Risks
Steroid Pulse Therapy	• Rapid suppression of intraocular inflammation. • Potentially quicker visual recovery. • Effective in preventing complications like retinal detachment.	• Increased risk of systemic side effects. • Potential for ocular complications like cataract formation and glaucoma. • Risk of infection due to immunosuppressive effects.
Oral Corticosteroids	• Gradual reduction of inflammation. • Lower risk of systemic side effects compared to pulse therapy.	• Risk of maternal hypertension and gestational diabetes. • Slower onset of action. • Requires prolonged therapy, increasing the risk of long-term side effects.
Immunosuppressive Agents	• Useful in steroid-sparing regimens. • May reduce the need for prolonged steroid use.	• Potential teratogenic effect • Potential for organ toxicity • Increased risk of infections. • Requires regular monitoring of blood counts and organ function.
Biologic Therapies	• Potential for rapid response • Useful in refractory cases.	• May cross the placenta • High cost. • Limited long-term safety data. • Potential for infusion reactions.

### Steroid pulse therapy

Steroid pulse therapy involves the administration of high doses of corticosteroids over a short period to rapidly suppress inflammation in patients with autoimmune disorders. This approach involves the intravenous infusion of high-dose corticosteroids, which may reduce toxicity compared with prolonged low-dose steroid treatments [7]. The preference for pulse therapy over conventional corticosteroid therapy lies in its ability to minimize side effects and reduce mortality rates [8]. It aims for quicker and stronger efficacy while decreasing the need for long-term steroid use, leveraging the paradoxical steroid-sparing effect achieved by high-dose administration. However, systemic infections and uncontrolled hypertension are contraindications for this therapy [7].

During pregnancy, steroids are used in the prevention of respiratory distress syndrome (RDS), intraventricular hemorrhage (IVH), chronic and acute adrenal insufficiency, congenital adrenal hyperplasia, and other complications, such as fetal heart block and antiphospholipid antibody syndrome [9]. Pulse therapy is effective in the management of nephrotic syndrome during pregnancy, where it can prevent preterm labor by improving maternal outcomes without the need for renal biopsy [10].

Commonly used corticosteroids in pulse therapy include methylprednisolone and dexamethasone. Methylprednisolone is typically administered at doses of 20–30 mg/kg (500–1000 mg/m²) per pulse (maximum dose of 1 g). Dexamethasone is given at doses of 4–5 mg/kg (100–200 mg) per pulse. The steroids are dissolved in 150–200 ml of 5% dextrose and infused slowly over 1–3 h to prevent hemodynamic issues from rapid infusions. Repeat doses are given every 24–48 h, with the frequency adjusted on the basis of the patient’s response [7].

Steroid treatment during pregnancy can affect the fetus differently depending on the type of steroid used. Fluorinated steroids such as dexamethasone and betamethasone are more likely to cross the placenta and reach the fetus in greater amounts, whereas nonfluorinated steroids such as prednisone and hydrocortisone cross the placenta to a much lesser extent and thus result in less fetal exposure. Hence, choosing the type of steroid in pregnant patients requiring high-dose therapy is vital to minimize any potential harm to the developing fetus [11].

Despite these benefits, steroid pulse therapy can lead to adverse effects such as hypertension, arrhythmias, hypokalemia, psychosis, increased susceptibility to infections, fluid retention, hyperglycemia, peptic ulcers, and osteoporosis [12]. Therefore, careful monitoring and specific indications are essential when this therapy is used [9]. In VKH, pulse steroid therapy has been highly effective, with a significant reduction in retinal detachment and improvement in visual acuity, with patients experiencing greater benefits than those receiving conventional therapies [13, 14].

Local treatment options should also be considered in managing VKH because of the risks associated with systemic steroid therapy and the need for high doses. In pregnancy, where systemic steroids may pose a significant risk, intravitreal steroid injections could be considered an alternative. Dexamethasone implants or triamcinolone intravitreal injections deliver steroids directly to the affected ocular tissues and minimize systemic exposure and side effects. While these local applications have shown promising results in controlling ocular inflammation and improving visual outcomes in VKH [4], their safety during pregnancy remains unclear. Therefore, these therapies should be used cautiously with careful consideration under the supervision of a multidisciplinary team to weigh the benefits and risks to both the mother and the fetus.

### Risks and long-term impact of steroid use on maternal and fetal health

Repeated courses of antenatal steroids increase the risk of maternal infections such as endometritis and chorioamnionitis. A single dose can increase maternal white blood cell counts and plasma glucose and amino acid levels. In the fetus, steroid exposure can reduce body movements, breathing activity, and heart rate variability, although it does not affect fetoplacental blood flow. Multiple courses may impair fetal growth and increase the risk of neonatal sepsis [15].

The long-term outcomes show mixed findings. A systematic review revealed that while extremely preterm infants experienced reduced neurodevelopmental impairment with corticosteroid exposure, late-preterm and full-term infants presented increased risks of neurocognitive and psychological disorders [16]. Additionally, the evidence on the associations of corticosteroids with oral clefts, low birth weight, and preeclampsia remains inconsistent, and their role in gestational diabetes is unclear [17].

Prolonged corticosteroid use during pregnancy significantly increases the risk of maternal complications such as preeclampsia and venous thromboembolism, as well as neonatal issues such as prematurity and low birth weight [18]. A Finnish cohort study also revealed an increased risk of mental and behavioral disorders in children exposed to antenatal corticosteroids, particularly those born at term, emphasizing the need for caution in late pregnancy use [19].

In a review of 32 cases, Matsuo T et al. concluded that corticosteroid therapy is both safe and effective for managing VKH during the third trimester of pregnancy. They reported that a course of steroid pulse therapy, followed by the tapering of oral prednisolone for more than 1.5 months, successfully treated the condition without complications. Most patients do not experience uveitis relapse after delivery. They recommended limiting the duration of prednisolone therapy and stressed the importance of closely monitoring the condition via OCT to ensure good outcomes for both mothers and fetuses [20].

### Managing VKH with multidisciplinary care

Effective management of VKH during pregnancy requires a multidisciplinary approach involving ophthalmologists, dermatologists, neurologists, rheumatologists, and obstetricians. Ophthalmologists are crucial for monitoring and managing the ocular manifestations of VKH, such as uveitis and retinal complications. Regular eye examinations and timely treatments are important to prevent vision loss and address flare-ups. Dermatologists play a key role in managing cutaneous symptoms, which affect patients’ quality of life. Neurologists are essential for handling neurological symptoms such as headaches and meningitis-like symptoms and ruling out other neurological issues during pregnancy. Rheumatologists provide expertise in managing the systemic aspects of VKH by coordinating immunosuppressive therapies and addressing interactions with pregnancy medications. Obstetricians oversee the overall health of both mothers and fetuses and coordinate care with other specialists to manage the impact of VKH and its treatments on pregnancy.

### Key areas for future research on VKH management during pregnancy

The management of VKH during pregnancy presents several challenges and opens multiple avenues for future research. One key area to explore is the long-term effects of steroid therapy on maternal and fetal health. While steroid pulse therapy has shown favorable short-term outcomes, its impact on neurodevelopment, endocrine function, and overall health in children exposed prenatally remains underexplored. Long-term follow-up studies of infants who are exposed to high-dose steroids during pregnancy are needed to identify any delayed or chronic health issues.

Another important aspect is determining the optimal steroid regimen for managing VKH during pregnancy. There is limited consensus on the best type, dosage, and duration of steroids. Future research should compare various steroid regimens, including different types of steroids (betamethasone vs. prednisolone) and administration methods (high-dose vs. pulse therapy), to establish the most effective and safest approach. Additionally, personalized risk assessment for VKH patients during pregnancy should be developed, as disease progression and risk factors can vary. Studying the mechanisms underlying VKH flare-ups during pregnancy and their interaction with pregnancy-related immune changes could lead to more targeted and effective treatment strategies.

## Conclusion

In conclusion, managing VKH during pregnancy requires a careful balance between effective treatment and minimizing risks to both the mother and fetus. Early use of corticosteroids is crucial for controlling symptoms with close monitoring of potential side effects. Future research should focus on understanding the long-term effects of steroid therapy on maternal and fetal health. Studies comparing different steroid regimens and treatment strategies are needed to establish the safest and most effective approach for pregnant patients.

## Data Availability

Not applicable, as no new data are generated.
